# Can higher calorie feeding protocols for eating disorder patients admitted to an adult hospital ward be safely implemented to reduce the length of stay? A scoping review

**DOI:** 10.1186/s40337-026-01669-9

**Published:** 2026-07-30

**Authors:** Jackson Taylor, Michael Salmon, Caitlin Rogash, Grace Branjerdporn

**Affiliations:** 1 Gold Coast University Hospital, Gold Coast Health, Queensland Health, Southport, QLD Australia; 2 Faculty of Health Sciences and Medicine, Bond University, Robina, QLD Australia

**Keywords:** Eating disorders, Anorexia nervosa, Adults, Inpatients, High calorie refeeding, Low-calorie refeeding, Refeeding syndrome, Length of stay

## Abstract

**Background:**

Hospital admissions for eating disorders are increasing, yet adult inpatient nutritional restoration often relies on conservative low-calorie refeeding to avoid refeeding syndrome (RFS). Evidence from younger populations suggests higher-calorie refeeding (HCR) may be safe and more efficient, but adult data is limited. This scoping review mapped the extent, characteristics and safety of HCR (≥ 1500 kcal/day) for people with eating disorders admitted to adult hospital wards.

**Methods:**

Five electronic databases were searched in July 2025 for studies published January 2012–June 2025. Eligible studies included adults with a diagnosed eating disorder, or mixed-age samples treated on adult wards, who commenced HCR on admission. Primary outcomes were RFS markers, particularly refeeding hypophosphataemia; secondary outcomes were length of stay (LOS), weight gain and change in body mass index (BMI). Three reviewers independently screened records and charted data, and findings were synthesised descriptively.

**Results:**

Of 778 records identified, 11 studies met inclusion criteria. Most were retrospective cohorts, with three quasi-experimental before–after designs, undertaken in adult specialist or general inpatient settings across several countries. Together they described 4874 patients exposed to HCR, usually starting at 1500–2000 kcal/day and advancing as tolerated. Definitions and monitoring of RFS varied widely. No study reported deaths or clinically defined RFS attributable to HCR. Biochemical hypophosphataemia affected up to around one-third of patients, was usually mild and managed with electrolyte supplementation. Four studies reported LOS; two found shorter admissions and faster weight gain with HCR compared with lower-calorie protocols.

**Conclusions:**

Across observational studies, initiating HCR for people with eating disorders on adult hospital wards appears feasible and can be delivered without an obvious increase in clinical RFS, while potentially reducing LOS and enhancing weight restoration when combined with close monitoring and supplementation. Heterogeneity in RFS definitions, refeeding protocols and outcome reporting, and the absence of randomised trials, limit confidence in these findings. Prospective studies using standardised RFS criteria and clearly defined HCR and comparator protocols are needed to guide adult-focused practice and policy.

**Supplementary Information:**

The online version contains supplementary material available at https://doi.org/10.1186/s40337-026-01669-9.

## Abstract (plain language summary)

People admitted to hospital for an eating disorder often need careful refeeding to restore nutrition. Many services still start with low-calorie feeding and increase slowly to avoid a rare but serious complication called refeeding syndrome (dangerous shifts in body electrolytes and fluids). However, newer studies—mostly in younger patients—suggest that starting with higher calories, alongside close blood-test monitoring and vitamin/mineral supplements, may be just as safe and could help people recover faster and leave hospital sooner.

This scoping review will map what is known about higher- versus lower-calorie refeeding on hospital wards treating adults with eating disorders. We will compare safety (refeeding syndrome and low blood phosphate), length of stay, and weight or body mass index changes. Because studies use different definitions and monitoring routines, we will record exactly how each study measured these outcomes so the findings can be compared fairly. The results help clinicians, patients, and services to have a clear overview of the evidence to support safe, timely, and effective inpatient nutrition care.

**Supplementary Information:**

The online version contains supplementary material available at https://doi.org/10.1186/s40337-026-01669-9.

## Background

Eating disorders (EDs) are severe psychiatric illnesses marked by persistent disturbances in eating behaviours and body image, often driven by weight and shape concerns [1]. Diagnoses include anorexia nervosa (AN), bulimia nervosa (BN), binge eating disorder (BED), and avoidant/restrictive food intake disorder (ARFID). EDs cause profound psychological distress and medical morbidity, with AN carrying the highest mortality of any psychiatric disorder, most often due to suicide or complications of prolonged malnutrition [2].

In Australia, up to 16% of adults will experience an ED in their lifetime [3]. Hospital admissions for ED increased by 91% between 2008 and 2018, with an average length of stay (LOS) of 21 days [4]. Many patients are admitted following prolonged starvation and are considered at risk of refeeding syndrome (RFS)—a potentially life-threatening cluster of electrolyte and fluid disturbances triggered by nutritional rehabilitation [5]. Concern about RFS has historically led services to adopt conservative “start low, go slow” refeeding protocols in the first 24–48 h of admission [5].

RFS was first described in the Minnesota Starvation Experiment, which documented metabolic changes during refeeding of semi-starved men [6]. Although influential, the study has ethical and methodological limitations and offers little guidance for contemporary ED care. International guidelines such as the National Institute for Health and Care Excellence (NICE) [7] define generic risk criteria, but these lack specificity for ED populations. More recent consensus statements, including the 2025 Australasian Society for Parenteral and Enteral Nutrition (AuSPEN) guidance, recommend against routine use of “start low, go slow” approaches and instead advocate progression to full nutritional requirements within the first 24–72 h, supported by structured monitoring and prophylactic supplementation [8]. Conservative low-calorie refeeding (LCR) aims to prevent RFS but may prolong catabolism, limit early weight gain, and extend LOS, potentially exacerbating bradycardia, hypotension, and fatigue that delay therapy engagement [9]. In contrast, higher-calorie refeeding (HCR) protocols, when combined with careful monitoring, appear equally safe and more effective [10]. In adolescents, a multicentre randomised trial found no difference in RFS, readmissions, or mortality between HCR and LCR, but HCR achieved faster weight restoration [11]. Systematic reviews in paediatric and young adult cohorts similarly report reduced LOS and no excess mortality with HCR, irrespective of disease severity [12].

By comparison, the adult evidence base is sparse. Australian clinical practice guidelines currently recommend a cautious “middle path” initiation of ~ 1400 kcal/day, reflecting uncertainty about how to balance RFS risk against the harms of underfeeding [13]. Previous adult studies suggest that higher caloric refeeding may be safe in eating disorder presentations with close medical monitoring if severely malnourished, defined as BMI < 15 kg/m^2^ [14]. A recent prospective observational study of eating disorder patients found that HCR supported shorter LOS and greater weight restoration, yet only three patients were diagnosed with severe malnutrition, with one of these patients requiring a deviation from the study protocol due to early features of refeeding syndrome [15]. Across adult studies, definitions of RFS, thresholds for biochemical abnormalities (e.g. hypophosphataemia), malnutrition status and body mass index (BMI) cut-offs are inconsistent, limiting synthesis and clinical translation compared to their paediatric and young adult counterparts [16].

Another key consideration is that many late adolescents and young adults with EDs transition from child and youth services into adult hospital wards during a period of changing legal status and autonomy. Treatment models and expectations often differ across these settings, and disengagement rates are high [17]. Because these individuals are frequently managed within adult inpatient units and subject to adult refeeding protocols, understanding the safety and outcomes of HCR in these environments is essential to ensure continuity of care can be provided across paediatric and adult service lines to support this transitional phase [18].

### Aims of the review

This scoping review aims to explore the existing evidence regarding HCR versus LCR protocols in hospital wards treating adults with eating disorders, including late adolescents and young adults admitted to a non-paediatric setting. Specifically, it seeks to answer the following research questions:

1. What is the existing evidence on the occurrence of RFS when initiating HCR in hospital wards treating adult eating disorder patients?
2. Does initiating HCR protocols in hospital wards treating adult eating disorder patients influence length of stay?
3. Does initiating HCR protocols in hospital wards treating adult eating disorder patients influence the rate of weight gain or BMI changes during admission?

## Method

### Protocol and registration

This scoping review was conducted following the Preferred Reporting Items for Systematic Reviews and Meta-Analyses Extension for Scoping Reviews (PRISMA-ScR) guidelines from the latest Joanna Briggs Institute (JBI) guidance for scoping reviews [19].

### Search strategy

The MEDLINE, CINAHL, American Psychological Association (APA) PsycArticles, ProQuest and Scopus electronic databases were used to conduct systematic literature searches.

The search strategy, including the databases, key search terms, and the number of articles found using this method, is shown in Supplementary 1. The search was undertaken during July 2025, limited to articles published between 1 Jan 2012 and 30 June 2025, as high-calorie refeeding remains a relatively novel approach. Potential additional studies were identified through snowballing; the reference lists of relevant systematic reviews, narrative reviews and meta-analyses identified in the “title/abstract” screening were searched in addition to the reference list of the Queensland Eating Disorder Service (QuEDS) guidelines.

### Selection criteria

#### Eligibility criteria

Eligibility was based on the PIO criteria: population, issues and outcomes to structure the review question and guide study selection.. Papers were included based on meeting the following criteria:

- Patients previously had a diagnosis of an eating disorder [anorexia nervosa, bulimia nervosa, binge eating disorder, avoidant and restrictive food intake disorder avoidant/restrictive food intake disorder (ARFID)].
- Patients were admitted to a hospital ward treating adult eating disorder patients (e.g. general medical, psychiatric, specialist inpatient unit for eating disorder treatment).
- Received HCR at a rate of ≥ 1500 calories/day initially as part of nutrition intervention.

### Population

Studies including individuals of any age or gender who received HCR due to an eating disorder with a diagnosis of AN, BN, EDNOS, OSFED, ARFID within a hospital setting requiring nutritional restoration. Studies were excluded if they were conducted on a unit that excluded eating disorder patients based on age (e.g. not providing care to those > 18 years of age).

### Issue

Commencing higher caloric refeeding (HCR) at a rate of ≥ 1500 calories/day for eating disorder patients admitted to an inpatient adult ward.

### Outcomes

Studies were eligible if they mentioned at least one primary measure related to refeeding syndrome markers during hospital admission. Examples of such markers included electrolyte derangement (i.e. hypophosphatemia, hypokalaemia, hypomagnesemia), peripheral oedema or a combination of these. Secondary outcomes of interest included length of stay, rate of weight gain and changes in admission and discharge Body Mass Index (BMI) were also reported, although this was not a necessary inclusion criteria.

### Study design

Studies included were randomised controlled trials, case-controlled studies, observational and cohort studies. Case studies, qualitative studies, abstracts of poster presentations, theses and grey literature were excluded.

#### Screening process

Title and abstract screening of all records was performed by three reviewers (JT, CR, MS), with one author as the first rater (JT), while second rater shared between the other two authors (MS, CR) using a web-based systematic review software (Covidence). Raters independently assessed the eligibility of the articles that were identified following the database search based on the eligibility criteria. Duplicates were removed within Covidence and manually checked. Records that appeared to meet the inclusion criteria based on title and abstract were retrieved for full-text review. Full-text screening was again performed independently by the same three reviewers, with discrepancies resolved through discussion and, where needed, majority consensus.

### Study selection

Data were extracted into an Excel spreadsheet created for the review by all three authors who completed full text screening, following an established framework for scoping reviews [20]. Data extracted included region of the study, type of study design, age of participants with an eating disorder, refeeding protocol and incidence of refeeding syndrome. Each of the reviewers independently recorded the data, discussed the results and continuously updated the data-charting form in an iterative process. Confirmation of data obtained from the included studies was reached on a majority basis. If no majority was reached, all three reviewers analysed the included paper together to reach a consensus. The main revisions through this process were charting if prophylactic supplementation was mentioned in the studies protocol, as this may have influenced how HCR was conducted.

## Results

### Study selection

**Fig. 1:**
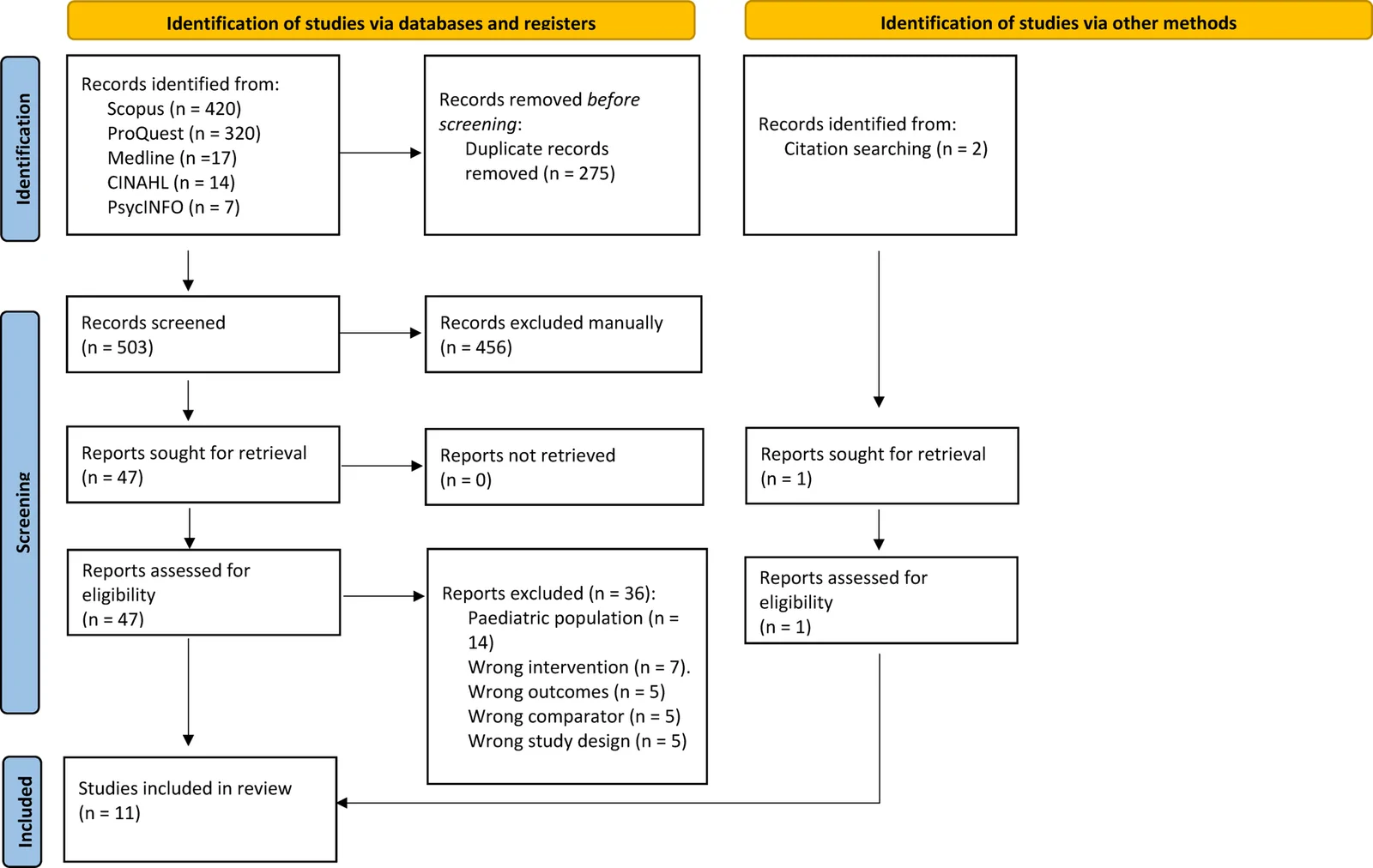
PRISMA study flow diagram showing the study selection process

The systematic database search yielded 778 articles. Once duplicates (*n* = 275 articles) were removed, the abstract and title of 503 articles were screened for inclusion in this review. Most of the articles (*n* = 456) were excluded due to irrelevancy. The remaining 47 full-text articles were screened for inclusion in this review. A further 36 articles were excluded for predefined reasons such as paediatric-only settings (*n* = 14) and incorrect interventions, such as initiating a LCR (*n* = 7). One article identified via citation chaining [21] was included, resulting in a total of eleven included papers. See Fig. 1 for the modified PRISMA flow diagram in which the search yields are presented [22].

### Summary of outcomes

Table 1 summarises the findings of the eleven included study designs, which were conducted in the United States, Germany, Australia, Canada and Switzerland. Most papers that met the inclusion criteria were observational and retrospective cohort studies, with three studies employing a quasi-experimental (before-after/natural-experiment) design [23, 25, 32]. The mean ages across the included studies demonstrated substantial variation depending on the population sampled. Mean ages ranged from 23.8 years [28] up to 32.5 years [29] for studies focused on adult eating disorder patients. Conversely, studies including paediatric and adolescent samples reported lower mean ages, such as 15.5 years [31], 17.3 years [25], and a median of 17 years [24]. The age range of all included studies was 8 years [24] and 66 years old [26]. Six of the eleven studies sampled patients only with an Anorexia Nervosa (AN) diagnosis [23, 25, 28, 30–32]. Eight studies were conducted in a specialised inpatient ward and settings for eating disorder patients, while three papers were conducted in a general hospital inpatient setting [21, 24, 27].

**Table 1:** Key methodological features and quantitative findings from included studies (*N* = 11)

Author (year) country	Study design and setting	Study aim/s	Participants (*n*) and age, y mean (SD)	Exclusion criteria	Refeeding protocol	Incidence of refeeding syndrome/hypophosphataemia	Rate of weight gain and length of stay	Relevant results
Haynos et al. (2016) [23] USA	Natural experiment design Inpatient eating disorder unit	1) Effect of change in caloric prescription plan on weight gain 2) Do standardised caloric plans impact LOS, discharge BMI, and maximum caloric prescription	Individualised (*n*=35) vs. standardised (*n*=35) caloric plan Subsample (*n*=20) for secondary analysis All participants’ diagnosis of AN Mean age: 24.51–26.34 years old (SD 6.4–7.3)	Discharge from inpatient unit prior to the second week of hospitalisation Medical illness unrelated to ED affected the ability to gain weight	Standardised approach: 1800 kcal/d for the first week of admission. Incremental 300–400 kcal increase every 2–3 days thereafter until 90% Ideal Body Weight (IBW) achieved Increases occurred independent of actual wt gain Individualised approach: Initially 1800 kcal/d An increase only occurred if wt failed to increase by 0.75lbs on weigh-in day and was achieved by consuming 400 kcal of food/250 kcal of liquid supplements. Did not exceed 4000 kcal/d	No notes of any cases RFS requiring medical care under either protocol RFS measured by presence of oedema throughout treatment	Mean Admission BMI 15.84–15.97 (SD 1.39–2.00) Aim to restore weight to 90% IBW (BMI 19.5) Weight gain 0.35 kg over the previous highest weight each week	Linear relationship of time to weekly rate of wt gain in standardised plan* Standardised plans gained wt faster compared to individualised* The standardised plan did not significantly affect LOS, discharge BMI, or maximum caloric prescription. Admission BMI as a covariate predicted time to wt restoration, presence of oedema and length of hospital stay*
Stranjord (2016) [24] USA	Retrospective chart review Inpatient hospital setting	Describe an inpatient nutritional insufficiency care path and compare treatment outcomes at an academic medical centre and a community hospital	Academic medical centre (*n*=51) vs. Community Hospital setting (*n*=39) Age - Range: 8–23 years. Median: 17 years.	Admissions <24 h Interhospital transfers between the sites	Initial median rate 2200(1750-2550) kcal. Increased by 250 (250–294) kcal in the academic centre and 305(250–525) kcal in the community hospital per day. Weight gain goal of 0.2 kg/day.	Hypophosphataemia defined as <0.8mmol/L Hypomagnesaemia defined as <0.7mmol/L Hypokalaemia defined as <3.5 mmol/L No clinical cases of refeeding, 1 patient had hypophosphatemia	Rate of wt gain 0.3 (0.1–0.4) kg/d in the academic centre and 0.2 (0.0–0.4) kg/d in the community hospital Median LOS was 3 days for both groups	Missed repeated laboratory studies during admission, likely underreported No weight criteria for discharge
Matthews et al. (2018) [21] Australia	Retrospective observational pre-test-post-test study Tertiary referral hospital	Compare the occurrence of RFS in LCR and HCR in medically compromised adult ED patients	*N*=119 Adults >18 years old at the time of inpatient admission with an ED diagnosis Mean Age: 27±9 years old	Admitted to ICU Renal conditions Pregnant LOS < 5 days	LCR: Initially 1000 kcal/d Progressed by 500 kcal/d every 3 to 4 days until the goal rate is achieved. Goal rate either individualised or 2400 kcal HCR: Initially 1500 kcal/d Progressed by 500 kcal/d every 48 h until the standardised goal rate of 3000 kcal/d was reached	No incidence RFS was reported in either group. Hypophosphatemia Definitions Present: <0.81mmol/L Mild: <0.68mmmol/L Critical: <0.32mmol/L	Admission BMI 14.9±2.6 in LCR and 17.2±3.0 in HCR Patients gained 1.8±2.4 kg with LCR and 2.3±2.3 HCR during acute admission. Length of stay or weight gain did not reach significance between groups	Improved adherence in HCR protocol compared to LCR*
Dalenbrook et al. (2020) [25] Germany	Single-centre study Inpatient eating disorder unit	Assess outcomes of an HCR protocol for adolescents with severe AN treated in a specialist eating disorder hospital	*N*=120 12–20 years old with primary diagnosis AN (any type) BMI < 15 kg/m2 Mean age: 17.3 ± 1.8 years	Pregnancy Bulimia nervosa schizophrenia or psychosis	Initial = 2000 kcal/d for all patients Aim for 700–1000 g weight gain/wk	No incidence of refeeding syndrome RFS, based on Rio et al., is defined as all the following criteria: Critically low serum electrolyte concentrations Potassium <2.5mmol/L Phosphate <0.32 mmol/L Magnesium <0.50 mmol/L Focused on serum phosphate concentrations as an indicator of impending refeeding syndrome	Baseline 35.6 kg (± 4.0 kg), Day 28 38.6 kg (± 4.2 kg) Average of 3 kg weight gain over 28 days LOS 38.4 (SD ± 28.4) days Mean BMI at baseline 13.1±1.1 kg/m^2^ to 16.7±1.8 kg/m^2^ at discharge*	HCR was not associated with refeeding even in severely malnourished adolescents with AN
Gibson et al. (2020) [26] USA	Retrospective chart review Inpatient eating disorder unit	Evaluation of medical complications observed in patients with extreme AN	*N*=281 Age – Range: 18–66 years. Median: 28 years Weighed < 65% Ideal Body Weight (IBW)	Hospitalised for < 72 h >65% IBW on admission	Initial caloric intake ranged 800–2450 kcal/d, with an average 1431 kcal/d for females and an average 1714 kcal/d for males The average caloric intake for females at the time of discharge was 3119 kcal/d (SD=733) and 3744 kcal/d for males (SD=829).	No deaths during medical stabilisation, *n*=20 (7%) required ICU transfer *n*=98 (35%) developed refeeding hypophosphatemia, with *n*= 46 developing critical hypophosphataemia Hypophosphataemia definitions: Mild: <0.87mmol/L Critical: <0.71mmol/L	Admission BMI mean 12.1 (range 7.5–15.7) Patients gained 2 kg/wk (SD =1.5) during acute admission. Average per cent IBW increased by 12% (SD=8.4) and average BMI increased by 2.5 kg/m2 (SD= 1.5) by time of discharge Median LOS = 22 days	No significant difference in weight gain/wk between genders (*p* =.84) Average nadir BMI between patients who developed RF hypophosphataemia (M= 11.8 kg/m2, SD = 1.4) compared to those who did not (M = 12.4 kg/m2, SD = 1.3)*
Gjoertz et al. (2020) [27] Switzerland	Retrospective chart review Inpatient hospital setting	Impact of introducing a clinical refeeding guideline for AN implemented in an inpatient setting	*N*=107 *n*=33 in the pre-guideline group; *n*=74 in the post-guideline group, aged 13–55 years, with a diagnosis of AN. Mean age at admission 27.6 ± 10.5 years (range 16.2– 53.3) in the first group, and 26.6 ± 11.5 years (range 13.6– 55.8) in the second group	*N*=18 excluded. Diagnosis of bulimia (*n* = 10), hospitalisation shorter than 7 days (*n* = 3), and a “respite” or “observation” type hospitalisation (*n* = 5).	Prior to Guideline: 75% of basic energy needs, energy added to ensure weight gain 500 g/wk Refeeding guideline: Stepwise approach. Energy needs defined as 35 kcal/kg/d for adults and 40–50 kcal/kg/d for adolescents depending on age and sex. In the first week, patients receive 50% of their individual basic energy needs at day 1, 75% at day 3, and 100% at day 6. During the second week, the basic energy needs are reached, and 300 kcal is added respectively every 3 days up to 1000 kcal, thus equaling 7000 kcal/wk, which corresponds to a gain of 1 kg/wk The aim was a systematic wt increase of >1 kg every week	Hypophosphataemia as per NICE Guidelines, defined as <0.8mmol/L Every patient received supplemental B1, folic acid, multivitamin, and Ca with Vit.D for 2-4weeks Oral replacement introduced if there are electrolyte deficiencies No incidence of refeeding syndrome	Mean weight gain of 7 ± 4.8 kg (range −1 to 17.3) vs. 5.8 ± 4.5 kg (range −1.7 to 2.0 kg) Mean BMI at admission was 14.3 ± 1.9 kg/m2 (range 11–19.8) before and 15 ± 1.8 kg/m2 (range 9.3–20.4) BMI at discharge was 17 ± 1.5 kg/m2 (range 13.7–20.7) before vs. 17.2 ± 1.6 kg/m2 (range 11.9–20) The mean length of hospitalisation decreased from 96.7 ± 48.3 days (range 9–180) to 62.2 ± 45.5 days (range 7–264)*	Only 6.3% of patients demonstrated optimal weight gain after the first 2 weeks prior to introduction compared with 41.8% after the guideline* In the first 2 weeks, 36.4% gained weight in the first group as opposed to 64.9% in the second group*, even though one-fifth in both groups demonstrated wt loss.
Koerner et al. (2020) [28] Germany	Retrospective naturalistic observation study Inpatient eating disorder unit	Impact of implementing an HCR protocol for adult AN ED patients on medical parameters such as changes in wt, blood phosphate levels and risk of RFS	*N*=103 Females aged 23.8±5.3 (range = 18–47) years with AN, BMI ≤13 kg/m2 and retention period ≥28 days	<18 years old BMI > 13 kg/m2 Other ED diagnoses	Initial oral meal plan 2000 kcal/d Up to >4000 kcal/d Calories adjustment individualised based on not meeting weight gain aim	Own criteria for RFS; Critical drop serum phosphate defined as <0.75mmol/L and critical deterioration of general condition (e.g. severe oedema, pericardial effusion, weakness) No patients experienced critical deterioration. No case of RFS reported. *N*=7 (6.2%) hypophosphatemia <0.75mmol/L, one person critical <0.5mmol/L on admission, 0 pathologic after 4 weeks 4-week admission, changes in LOS were not measured in the study	Aimed for wt increase 0.7–1.0 kg/wk Wt increased on average 1.0±0.5 kg/wk and by a total of 4.2±2.0 kg over four weeks* Total increase 1.4±0.8 BMI units in four weeks#	Individual energy intake and adherence to dietary recommendations were not recorded
Richardson et al. (2021) [29] Canada	Retrospective chart review Inpatient eating disorder unit	Review charts of all inpatients with an ED admitted to the program. Primary objective = assess if medical parameters i.e. presence of hypophosphataemia at admission were related to clinical outcome	*N*=288 admissions *n*=235 admissions met the ‘significantly’ medically compromised group n=17 met ‘moderately’ compromised group 32.5 (SD = 11.4) years	None. Those who didn’t fit significant or moderate groups were assigned to ‘other’ (*n*=36)	Changes in increases per week of treatment from an additional 200–300 kcal/wk to ~600 kcal/wk in 2019. Week 1 prescribed 1682 (SD = 352) kcal in the significant group, and weekly increases up to Week 4 2538 (SD = 502) kcal in the significant group	Hypophosphataemia defined as <0.80mmol/L Those who developed hypophosphataemia had lower admission BMI (BMI 14.1 SD = 3.58 vs. 16.4, SD = 3.51)* Only hypophosphataemia was found to be a statistically significant predictor of ICU admission. However, no patients were transferred ICU due to this.	Mean BMI at admission = 14.8 (SD = 1.8), range = 10.2–18.4 kg/m2 Mean BMI at 4 weeks = 16.1 (SD=1.9), range = 11.2–21.3 kg/m2 Rate of weight gain was not commented on. Mean LOS = 38.4 (SD = 28.4) days, (range = 1–126 days, 1 outlier 253 days). No data on changes in LOS	Refeeding syndrome was not discussed. Incidence of hypophosphatemia was key measure. BMI at admission was associated with BMI at 4 weeks*
Cuntz et al. (2022) [30] Germany	Retrospective observational study Inpatient eating disorder unit	1) Risk estimation based on labs to judge the overall health status in AN and RFS 2) Use risk estimation to evaluate health status during RFS using a HCR protocol	Sample 1 *n*=3230 Sample 2 *n* = 520 Mean age 22.5 years, range 12–73 years old, with AN and BMI <13 kg/m2 Retention period ≥28 days	Adm <28d, missing complete data set <18yrs	Initial 2000 kcal Up to 4000 kcal Calories adjusted according to wt development to aim for an increase in BW 0.7–1.0 kg/wk. Aim for 700–1000 g/wk gain. 100 g per day	Hypophosphataemia definition Critical: <0.5mmol/L *N*=16 hypophosphataemia (11 <0.75mmol/L, 5 <0.5mmol/L) at start of admission with mean BMI 11.5 kg/m2 Serum PO4 increased under supplementation. No cases of hypophosphataemia occurred, except for those with low PO4 at admission. After 2 weeks, all but *n*=1 was within normal range.	Mean weight gain first week 2.1 kg, followed by min. 700 g in the next 3 weeks. Mean total weight gain was 4.15 kg ± 1.96 kg Mean BMI 15.4 kg/m2 (range 9.4–26.8) – some had been in remission. Mean BMI admission to 28 days 1.7±0.9 kg/m^2^* No data on LOS	Negative correlation with RFS risk and BMI*^+^
Kells et al. (2022) [31] USA	Retrospective chart review Inpatient eating disorder unit	Identify individual factors and clinical interventions that contribute to the development of RH for adolescents and young adults admitted to a general medical unit with AN	*N*=300 10–24 years. Mean age = 15.5 years with an AN diagnosis (any)	BN, BED, Severe Mental Illness (schizophrenia, schizoaffective disorder) Other feeding methods besides oral and NG Medical or psychiatric hospitalisation within the last 14 days	1000-3000 kcal/d, most increased by 250 kcal/d Initial calorie prescription: mean 1714 kcal/d (SD = 324.2 kcal/d) Aim for 0.2 kg/d wt gain	RH defined as serum phosphate <0.90mmol/L *N*=86 (28.7%) reported hypophosphataemia at some point during hospital stay. Mean day occurrence = 3.9 (SD = 3.4) Phosphorous Prophylaxis 250 mg BD + MV Predictors of RH = Serum Mg, K, NG feeding, wt gain during adx, age on adx Mg nadir and BMI on adx predictors of PO4 nadir despite standardised PO4 supplementation	Average wt gain during hospitalisation = 1.8 kg (SD = 1.5 kg) Mean LOS = 7.4 days (SD = 5.9 days)	Mean BMI on admission 16.3 (SD = 2.6), did not measure discharge BMI. Supportive of prophylactic PO4 supplementation Measured RH, not refeeding syndrome
Staab et al. (2022) [32] Canada	Cohort natural quasi-experimental design Inpatient and day hospital eating disorder unit	Change of refeeding protocol and impact on wt gain, LOS and RFS occurrence in inpatient and day program Secondary aims to look at safety indicators i.e. electrolyte disturbances and supplementation, RFS, transfer to internal medical ward, completion of program	*N*=326 inpatients BMI <18 kg/m2 Mean age HCR 28.83 (SD 11.01) years old and LCR 29.27 (SD 11.8) years old Admissions were treated independently for patients admitted more than once during the study period	Other ED Dx (ARFID, EDNOS, BN) Inpatient stay <7 days Required NGT/modified meal plan based on other medical diagnoses	LCR: initially 1500-1600 kcal/d and gradually increased by 300 kcal each week up to maintenance level 2100-2300 kcal/d. Calorie level maintained as long as pt gaining >1 kg/week. If not, further increased by 300 kcal/week. HCR: Initially 1600-1800 kcal/day and increased by 600 kcal/week to 2800 kcal/day by day 21, regardless of wt gain. If patients failed to gain 1 kg/week, further increase 300–400 kcal/day	Hypophosphataemia defined as present if below the reference range 0.87–1.52mmol/L. Mild = 0.65-0.91mmol/L Mod = 0.32-0.64mmol/L Severe = <0.31mmol/L Severe Hypokalaemia = <2.5mmol/L Severe Hypomagnesaemia = <0.50mmol/L Hypophosphataemia was not significantly different between slow and rapid RF protocols after admission. No cases of severe hypophosphataemia in either group. Total cases mild and moderate hypophosphataemia not sig. between groups No deaths or cases RFS with either protocol over the 14-year period Amount PO4 supplements used to treat PO4 deficiencies was higher in the slow protocol compared to the rapid protocol	Mean admission weight 39.88 kg (SD 5.79) Mean admission BMI 14.88 (SD 1.38) HCR gained 0.21 kg/wk more compared to LCR. Higher rate of wt gain in IPU setting (1.30 kg/week compared to 1.08 kg/week)^#^ Average LOS = 40 days in Rapid RF Protocol compared to Average LOS = 53 days in slow RF Protocol. LOS 21 days shorter for pts on HCR vs. LCR*	No statistically significant differences between the two cohorts Reduction in mean days to reach target BMI 19.5 kg/m^2^ between LCR (81.61 SD 30.20) and HCR (59.04 SD 24.52)* Electrolyte supplementation was used more in LCR vs. HCR participants.

AN: anorexia nervosa; BMI: body mass index; ED: eating disorder; HCR: higher calorie refeeding; IBW: Ideal body weight; K: potassium; LCR: low calorie refeeding; LOS: length of stay; Mg: magnesium; PO4: phosphate; RFS: refeeding syndrome; RH: refeeding hypophosphataemia; SD: standard deviation; Wt: weight

^*^Significance (*p* <.001)

^#^Significance (*p* <.05)

^+^Spearman correlation (*r* =.41)

Exclusion criteria varied across study designs by diagnosis, duration of hospitalisation, weight status, and concurrent medical conditions. Five papers focused solely on AN by excluding other eating-disorder diagnoses [25, 27, 28, 31, 32], while three included broader diagnoses [21, 24, 29]. Length-of-stay (LOS)/retention-period exclusions were common: <24 h [24], < 72 h [26], < 5 days [21], and < 7 days [23, 27, 32]. Two cohorts required LOS ≥ 28 days [28, 30], and others reported no explicit LOS-related exclusion [25, 29, 31]. Additional exclusions included pregnancy and non-ED medical comorbidities [21, 23]. In all included studies, female participants were proportionally higher than males, and Koerner et al. [28] reported no males meeting the BMI < 13 kg/m² inclusion threshold.

Weight-status criteria across the eleven studies varied by function (inclusion vs. exclusion) and metrics, such as BMI, percentage median BMI (%mBMI) for patients, calculated using the 50th BMI percentile for age and sex, and percentage of IBW, calculated using the Hamwi method [35]. An explicit exclusion ceiling was used in Gibson et al. [26] (> 65% IBW excluded), and a severe-underweight entry threshold in Koerner et al. [28] (BMI < 13 kg/m²). Youth/mixed-age cohorts operationalised severity using the percentage median BMI (%mBMI) for patients, calculated using the 50th BMI percentile for age and sex [25, 27, 29, 31], while several adult or mixed cohorts reported no explicit weight-based exclusion and relied on clinical admission pathways [21, 23, 30, 32]. Where specified, some cohorts described very-low BMI inclusion bands (e.g., < 15 kg/m²) to characterise baseline severity.

The observational study by Cuntz et al. [30] was the largest, with 3230 admissions in a 15-bed specialised inpatient ward for eating disorder patients across four years. The smallest study included was that of Haynos et al. [23], which initially involved 70 eating disorder patients in their quasi-experimental design and a subsample of 20 for secondary analysis of RFS occurrence. Across the eleven included studies, a total of 4,874 patients were analysed for incidence of RFS.

### Study findings

All eleven studies reported on hypophosphataemia, with eight papers measuring refeeding hypophosphatemia (RH), while one measured the incidence of refeeding by the presence of oedema only [23]. Of the eight studies reporting on RH, there was heterogeneity between the definitions and severity thresholds (i.e., mild, critical). The cut-off for RH ranged between 0.75mmol-0.91mmol/L, while “critical” RH ranged from 0.31-0.71mmol/L.

Two studies included in this analysis utilised the same cut-offs based off establishing their own criteria for refeeding syndrome occurrence [28, 30], while two studies defined it based on Rio et al. [33] criteria [25, 32] and four studies cited criteria based off NICE guidelines [7, 21, 24, 27, 29]. The other two studies with a lower mean age sample defined hypophosphataemia as < 0.9mmol/L in alignment with adolescent guidelines [26, 31]. Only two of the eleven studies reported cases of RH [26, 31], with Gibson et al. [26] reporting it occurred in 35% of their sample (5 males and 93 females), with 47% of these patients (3 males and 43 females) meeting their criteria for critical hypophosphataemia. There were no deaths or incidence of refeeding syndrome reported in all included studies. When accounting for BMI, four papers found that a lower admission BMI was significantly (*p* <.001) associated with an increased risk in predicting RH [23, 26, 29] with Cuntz et al. [30] reporting a highly significant and negative correlation (Spearman correlation 0.41, *p* <.000) between the two variables. No other covariate was found to reach significance in predicting RH across the eleven studies.

Only four studies reported LOS as an outcome [21, 23, 27, 32]. Of these, Gjoertz et al. [27] and Staab et al. [32] were the only studies to reach significance, reporting mean LOS reductions of 34 and 21 days for eating disorder patients on HCR compared with LCR, respectively (*p* <.001). Haynos et al. [23] and Matthews et al. [21] additionally reported higher adherence to treatment under standardised HCR protocols than under individually tailored HCR plans (reporting metrics detailed in Table 1).

Two studies compared LCR and HCR protocols [21, 32], while the remaining studies only focused on an HCR protocol. No standard definition of “LCR” or “HCR” was shared across studies. Matthews et al. [21] compared initiating 1,000 kcal/d (LCR) versus 1,500 kcal/d (HCR), while Staab et al. [32] compared initiating at 1500–1600 kcal/d versus 1600–1800 kcal/d. The highest initial calories prescribed as a standardised approach was 2000 kcal/d [25, 28, 30], with the upper reported goal of 7000 kcal/d [27], and other goals ranging 2,500–4,000 kcal/d. One study did not include a fixed feeding protocol, providing a range and mean of initial and discharge calorie intake based on eating disorder patients gaining ~ 2 kg/week [26]. This study also stratified initial and discharge calories between male and female eating disorder patients and found no significant sex difference in weight gain per week [26].

Most papers (90%) reported on at least one secondary outcome (rate of weight gain, changes in admission and discharge BMI), with only one study focusing instead on comparing HCR treatment between a medical centre and community hospital rather than differences in these parameters [24]. Of the nine studies that reported weight gain, five reported higher mean weekly gains under HCR (0.7–2.1 kg/week). One study reported no significant difference in weight gain within the first week [21]. Five studies reported no difference in weight-gain rate between minors and adults [24, 25, 27, 30, 31]. The quasi-experimental study by Staab et al. [32] reported a shorter time to reach target BMI under HCR versus LCR (median 59 vs. 81 days; *p* <.001) and a higher mean rate of weight gain by 0.21 kg/week under HCR.

## Discussion

Across predominantly retrospective and quasi-experimental cohorts, observational evidence suggests that HCR is associated with faster in-hospital progress—shorter length of stay and greater weekly weight gain—without a clearly increased rate of clinical RFS and with variably reported but generally manageable rates of RH. Taken together, the eleven studies suggest that, in supervised inpatient settings with electrolyte monitoring and supplementation protocols, initiating at ≥ 1,500–2,000 kcal/d and advancing progressively may offer operational advantages to hospital and health services compared with LCR. Notably, clinical RFS events were uncommon, and no deaths were reported; however, follow-up was short, and studies were not powered for rare safety outcomes.

LCR protocols for eating disorder patients have been recommended substantially, starting from ~ 1,200 kcal/d or lower and incrementing slowly [23]. This clinical paradigm of ‘start low, go slow’ was rationalised to minimise the risk of RFS occurrence and limit preventable deaths. Claims that LCR “succeeded” because few RFS cases were reported should be interpreted cautiously given historical under-detection and varied definitions [5, 7]. However, the increasing prevalence of EDs in Australia and the resulting pressures these place on healthcare systems give rise to the need to seek clinical practices that are both safe and efficient [4]. Within these constraints, this scoping review signals that HCR can be delivered safely in specialist inpatient settings with protocolised monitoring; nevertheless, certainty is limited by non-randomised designs, baseline imbalances, and measurement heterogeneity.

While all studies reported on refeeding, many differing outcomes to determine and define occurrence were reported between studies. Most studies quantified biochemical RH (low serum phosphate), not clinical RFS, which aligns with previous literature [16]. However, the manifestations of RFS remain non-unanimous and non-specific, so using RH alone as a surrogate for RFS risks misclassification [5]. Where examined, associations between RH and clinical RFS were inconsistent and generally underpowered; thus, no firm linkage can be made in these cohorts. Alternative and adjunctive markers such as oedema have been proposed but were applied inconsistently [23, 28].

Phosphate supplementation was reported by nine papers to varying degrees in indication, dosage, and timing [21, 24–28, 30–32], with only Staab et al. [32] reporting more frequent use in LCR than HCR (not statistically significant). High variations in reported RH likely reflect different electrolyte correction strategies and dosages, a well-documented finding in adolescent EDs literature [10, 12]. Standardising sampling windows (e.g., timing of first 72 h draws), RH thresholds (adult vs. adolescent), and prophylactic versus reactive supplementation would improve comparability and enable data pooling in future studies.

Across studies reporting RH, samples differed in disease severity, with Gibson et al. [26] mean admission BMI 12.1 kg/m² compared to Kells et al. [31] 16.3 kg/m². While Gibson et al. [26] included severe eating disorder patients, it also had a higher RH cut-off than other included studies. Multiple studies reported an inverse association between lower admission BMI and RH risk, but effect sizes and significance varied, and not all analyses were adjusted for confounders [30]. No deaths occurred and reported clinical RFS severity was minimal, albeit with limited follow-up and inpatient-bound outcomes. Unlike adolescent eating disorder patients literature, adult cohorts lack higher-quality prospective or randomised studies with longer follow-up [11].

LOS appeared shorter in some HCR cohorts, including severe eating disorder patient admissions [32], consistent with paediatric and adolescent focused studies [9]. Unfortunately, significant variations in the length of admissions and follow-up times between included studies and baseline case-mix differences make generalisations difficult. Also, severe eating disorder patient admissions may require more prolonged hospital admission due to medical complications irrespective of RFS risk or occurrence, introducing confounding by indication [34].

### Limitations and strengths of study

The strengths of this review were that studies included severely malnourished eating disorder patients across adolescent, young adult and adult populations [26, 28, 30, 32], defined by including participants with a BMI < 15 kg/m² [1]. Furthermore, no consistent association between higher starting calories and increased clinical RFS was observed across studies, despite large variations. This result aligns with previous adolescent studies in eating disorder patients that HCR can be delivered safely in mild to moderate disease severity [10, 11].

While the quality of studies was not rigorously tested as part of this scoping review, most evidence from included studies was derived from retrospective or observational study designs. These kinds of studies are subject to biases, such as selection bias. Confounding by indication/era (e.g., lower admission BMI, centre protocols, and admission pathways), measurement heterogeneity (RH/RFS definitions and sampling schedules), and incomplete reporting of intervention fidelity (starting-calorie adherence and advancement) are likely to influence observed effects. Selection bias may have occurred because patients perceived as high-risk RFS were assigned to an initially lower calorie prescription where standardised approaches were not used. Electrolyte supplementation (indication/dose/timing) may also confound RH incidence. Furthermore, one included study accounted for 66% of all included study subjects [30], a significant imbalance that may affect the generalisability of these findings.

### Evidence gaps and future research directions

HCR may decrease LOS compared to LCR in eating disorder patient presentations to adult medical services, particularly those with severe AN (admission BMI < 15 kg/m²) in tertiary hospital settings. This potential benefit should be confirmed in prospective designs and interpreted alongside resource and patient-centred outcomes. Given that current RFS criteria places substantial emphasis on low BMI, future work should examine whether multivariable risk tools outperform single-threshold approaches for predicting clinically meaningful RFS.

There is no universal definition of RFS, with significant variations in criterion markers between studies, making data synthesis difficult. Consensus-building efforts between researchers, clinicians, and policymakers are needed to standardise RFS criteria and RH thresholds across both adult and young adult services that are age and developmentally inclusive.

Future studies should explore the role of electrolyte supplementation, particularly in severe ED presentations where admission BMI is < 15 kg/m². Due to heterogeneity and some studies not reporting on its occurrence, it is unclear to what degree this may influence the risk of RFS and occurrence, as well as the different approaches to supplementation (i.e., prophylactic versus reactive). Prospective protocols should pre-register supplementation triggers, dosing, and monitoring to reduce performance and detection bias.

While no deaths were reported in any of the included studies, there was minimal follow-up time in the included studies. Longer-term follow-up is required to assess morbidity, readmissions, and functional outcomes, and to evaluate any risks potentially associated with accelerated weight-gain targets (e.g., > 2 kg/week).

## Conclusion

This scoping review has synthesised and summarised evidence regarding the clinical practice of HCR in adult eating disorder patients within a hospital setting. Regarding safety, current observational evidence indicates that HCR can be implemented without a clear increase in clinical RFS in adult eating disorder patients compared to the usual practice of LCR. However, the generalisation of results is limited due to considerable heterogeneity in how RFS is defined and measured. While HCR approaches are beginning to be adopted in paediatric and adolescent eating disorder patient settings, limited high-quality studies, such as RCTs, currently exist in adult eating disorder patients, with no such studies identified and included in this review. The quality of included studies was limited due to the majority being retrospective or observational in design. Accordingly, stronger prospective evidence with standardised RH/RFS definitions, electrolyte protocols, and adherence reporting is required to inform practice and policy. Furthermore, future studies involving longer follow-up periods would be beneficial to determine if there are any other covariates in adult patients with EDs who receive HCR in response to accelerated weight gain targets and potentially decreased LOS.

## Supplementary Information

Supplementary Material 1

Supplementary Material 2

## Data Availability

- All the data generated or analysed during this study are included in this published article and its supplementary information files. https://doi.org/10.1079/searchRxiv.2026.01215.

## References

[1] American Psychiatric Association ib. Diagnostic and statistical manual of mental disorders: DSM-5-TR. Fifth edition, text revision. ed. Washington, DC: American Psychiatric Association Publishing; 2022.

[2] Auger N, Potter BJ, Ukah UV, Low N, Israël M, Steiger H, et al. Anorexia nervosa and the long-term risk of mortality in women. World Psychiatry. 2021;20(3):448–9. 10.1002/wps.20904 PMC8429328 34505367

[3] Hay P, Girosi F, Mond J. Prevalence and sociodemographic correlates of DSM-5 eating disorders in the Australian population. J Eat disorders. 2015;3(1):19. 10.1186/s40337-015-0056-0 PMC4408592 25914826

[4] Nguyen M, Allison S, Looi JCL, Bastiampillai T. Increasing hospital admission rates for anorexia nervosa amongst young women in Australia from 1998 to 2018. Australasian psychiatry: Bull Royal Australian New Z Coll Psychiatrists. 2022;30(4):462–71. 10.1177/10398562221077890 35225008

[5] da Silva JSV, Seres DS, Sabino K, Adams SC, Berdahl GJ, Citty SW, et al. ASPEN consensus recommendations for refeeding syndrome. Nutr Clin Pract. 2020;35(2):178–95. 10.1002/ncp.10474 32115791

[6] Keys A, Brožek J, Henschel A, Mickelsen O, Taylor HL. The biology of human starvation. University of Minnesota Press; 1950.

[7] Nutrition support for. adults: oral nutrition support, enteral tube feeding and parenteral nutrition. London: National Institute for Health and Care Excellence (NICE); 2017. 31999417

[8] Matthews-Rensch K, Blackwood K, Lawlis D, Breik L, McLean C, Nguyen T, et al. The Australasian Society of Parenteral and Enteral Nutrition: consensus statements on refeeding syndrome. Nutr Diet. 2025;82(2):128–42. 10.1111/1747-0080.70003 PMC11973624 40090863

[9] O’Connor G, Nicholls D. Refeeding Hypophosphatemia in Adolescents With Anorexia Nervosa: A Systematic Review. Nutr Clin Pract. 2013;28(3):358–64. 10.1177/0884533613476892 PMC4108292 23459608

[10] Garber AK, Sawyer SM, Golden NH, Guarda AS, Katzman DK, Kohn MR, et al. A systematic review of approaches to refeeding in patients with anorexia nervosa. Int J Eat Disord. 2016;49(3):293–310. 10.1002/eat.22482 PMC6193754 26661289

[11] Golden NH, Cheng J, Kapphahn CJ, Buckelew SM, Machen VI, Kreiter A, et al. Higher-Calorie Refeeding in Anorexia Nervosa: 1-Year Outcomes From a Randomized Controlled Trial. Pediatr (Evanston). 2021;147(4):1. 10.1542/peds.2020-037135 PMC8015147 33753542

[12] Mosuka EM, Murugan A, Thakral A, Ngomo MC, Budhiraja S, St. Victor R. Clinical outcomes of refeeding syndrome: a systematic review of high vs. low-calorie diets for the treatment of anorexia nervosa and related eating disorders in children and adolescents. Cureus. 2023;15(5):e39313. 10.7759/cureus.39313 PMC10281854 37351245

[13] Hay P, Chinn D, Forbes D, Madden S, Newton R, Sugenor L, et al. Royal Australian and New Zealand College of Psychiatrists clinical practice guidelines for the treatment of eating disorders. Aust N Z J Psychiatry. 2014;48(11):977–1008. 10.1177/0004867414555814 25351912

[14] Redgrave GW, Coughlin JW, Schreyer CC, Martin LM, Leonpacher AK, Seide M, et al. Refeeding and weight restoration outcomes in anorexia nervosa: Challenging current guidelines. Int J Eat Disord. 2015;48(7):866–73. 10.1002/eat.22390 25625572

[15] Salter F, Singh U, Kerr D, Zhao Y, Jeffery E. A prospective observational study examining weight and psychosocial change in adolescent and adult eating disorder inpatients admitted for nutritional rehabilitation using a high-energy re-feeding protocol. J Eat disorders. 2024;12(1):58–14. 10.1186/s40337-024-01015-x PMC11094855 38745266

[16] Kraft MD, Btaiche IF, Sacks GS. Review of the refeeding syndrome. Nutr Clin Pract. 2005;20(6):625–33. 10.1177/0115426505020006625 16306300

[17] Ragnhildstveit A, Tuteja N, Seli P, Smart L, Uzun N, Bass LC, et al. Transitions from child and adolescent to adult mental health services for eating disorders: an in-depth systematic review and development of a transition framework. J Eat disorders. 2024;12(1):36–17. 10.1186/s40337-024-00984-3 PMC10921655 38454528

[18] Wade TD. A systematic review: solutions to problems caused by age transition between eating disorder services. Eur Eat Disord Rev. 2023;31(2):247–57. 10.1002/erv.2945 35951534

[19] Peters MDJ, Marnie C, Tricco AC, Pollock D, Munn Z, Alexander L, et al. Updated methodological guidance for the conduct of scoping reviews. JBI Evid Implement. 2021;19(1):3–10. 10.1097/XEB.0000000000000277 33570328

[20] Arksey H, O’Malley L. Scoping studies: towards a methodological framework. Int J Soc Res Methodol. 2005;8(1):19–32.

[21] Matthews K, Hill J, Jeffrey S, Patterson S, Davis A, Ward W, et al. A Higher-Calorie Refeeding Protocol Does Not Increase Adverse Outcomes in Adult Patients with Eating Disorders. J Acad Nutr Dietetics. 2018;118(8):1450–63. 10.1016/j.jand.2018.01.023 29656932

[22] Page MJ, McKenzie JE, Bossuyt PM, Boutron I, Hoffmann TC, Mulrow CD, et al. The PRISMA 2020 statement: an updated guideline for reporting systematic reviews. BMJ. 2021;372:n71. 10.1136/bmj.n71 PMC8005924 33782057

[23] Haynos AF, Snipes C, Guarda A, Mayer LE, Attia E. Comparison of standardized versus individualized caloric prescriptions in the nutritional rehabilitation of inpatients with anorexia nervosa. Int J Eat Disord. 2016;49(1):50–8. 10.1002/eat.22469 PMC4717916 26769581

[24] Strandjord SE, Sieke EH, Richmond M, Khadilkar A, Rome ES. Medical stabilization of adolescents with nutritional insufficiency: a clinical care path. Eating Weight Disord. 2016;21(3):403–10. 10.1007/s40519-015-0245-5 26597679

[25] Dalenbrook S, Naab S, Garber A, Correll C, Voderholzer U, Haas V. Outcomes of a standardized, high-caloric, inpatient re-alimentation treatment protocol in 120 severely malnourished adolescents with anorexia nervosa. J Clin Med. 2022;11(9):2585. 10.3390/jcm11092585 PMC9105338 35566710

[26] Gibson D, Watters A, Cost J, Mascolo M, Mehler PS. Extreme anorexia nervosa: medical findings, outcomes, and inferences from a retrospective cohort. J Eat disorders. 2020;8(1):25–10. 10.1186/s40337-020-00303-6 PMC7310519 32582446

[27] Gjoertz M, Wang J, Chatelet S, Monney Chaubert C, Lier F, Ambresin AE. Nutrition Approach for Inpatients With Anorexia Nervosa: Impact of a Clinical Refeeding Guideline. JPEN J Parenter Enter Nutr. 2020;44(6):1124–39. 10.1002/jpen.1723 31742725

[28] Koerner T, Haas V, Heese J, Karacic M, Ngo E, Correll CU, et al. Outcomes of an accelerated inpatient refeeding protocol in 103 extremely underweight adults with anorexia nervosa at a specialized clinic in Prien, Germany. J Clin Med. 2020;9(5):1535. 10.3390/jcm9051535 PMC7291118 32438760

[29] Richardson C, Huniewicz P, Paslakis G. Retrospective analysis of hypophosphatemia rates and other clinical parameters in patients with eating disorders. Eur Eat disorders Rev. 2021;29(2):193–203. 10.1002/erv.2810 33247867

[30] Cuntz U, Körner T, Voderholzer U. Rapid renutrition improves health status in severely malnourished inpatients with AN - score‐based evaluation of a high caloric refeeding protocol in severely malnourished inpatients with anorexia nervosa in an intermediate care unit. Eur Eat disorders Rev. 2022;30(2):178–89. 10.1002/erv.2877 PMC9299673 34889001

[31] Kells M, Gregas M, Wolfe BE, Garber AK, Kelly-Weeder S. Factors associated with refeeding hypophosphatemia in adolescents and young adults hospitalized with anorexia nervosa. Nutr Clin Pract. 2022;37(2):470–8. 10.1002/ncp.10772 PMC8962676 34494697

[32] Staab R, Campagna J, Ma J, Sengar A. Rapid refeeding in anorexia nervosa: A dialectic balance. Int J Eat Disord. 2022;55(5):653–63. 10.1002/eat.23698 PMC9315141 35332954

[33] Rio A, Whelan K, Goff L, Reidlinger DP, Smeeton N. Occurrence of refeeding syndrome in adults started on artificial nutrition support: prospective cohort study. BMJ open. 2013;3(1):e002173. 10.1136/bmjopen-2012-002173 PMC3549252 23315514

[34] Garber AK, Cheng J, Accurso EC, Adams SH, Buckelew SM, Kapphahn CJ, et al. Short-term Outcomes of the Study of Refeeding to Optimize Inpatient Gains for Patients With Anorexia Nervosa: A Multicenter Randomized Clinical Trial. JAMA Pediatr. 2021;175(1):19–27. 10.1001/jamapediatrics.2020.3359 PMC7573797 33074282

[35] Hamwi G. Therapy: changing dietary concepts. Diabetes mellitus: diagnosis Treat. 1964;1:73–8.

