# Supplementary material for: Can higher calorie feeding protocols for eating disorder patients admitted to an adult hospital ward be safely implemented to reduce the length of stay? A scoping review
Source: J Eat Disord. 2026 Jul 30;14:260. doi: 10.1186/s40337-026-01669-9 (PMC13741680; doi:10.1186/s40337-026-01669-9)
Supplement: Supplementary file 1 [file 40337_2026_1669_MOESM1_ESM.docx]

### Supplementary 1: Database search strategy

| ***Database*** | ***Search terms*** | ***No. articles*** |
| --- | --- | --- |
| **Medline** | "Feeding and Eating Disorders+" [MeSH] OR "Eating disorder*" OR anorex* OR “anorexia nervosa” OR bulimia OR “binge-eating disorder” | N = 11 |
| AND | "Inpatients" [MeSH] OR Inpatient OR “in hospital care” OR “in hospital treatment” OR treatment OR “mental health unit*” OR hospitalisation OR "acute admission" OR acute |  |
| AND | "Energy Intake+" [MeSH] OR "Protein-Energy Malnutrition" [MeSH] OR “Caloric feeding protocol” OR “feeding protocol” OR “nutrition supplementation” OR “nutrition restoration” OR "enteral nutrition" OR “energy intake” |  |
| AND | “Refeeding Syndrome” [MeSH] OR “refeeding syndrome” OR “hypophospha*” OR “phosphate” |  |
| AND | "Length of Stay" [MeSH] OR "length of stay" OR "length in hospital" OR duration |  |
| Limiters: | 2012-2025; English; peer reviewed |  |
| **CINAHL** | "Eating Disorders+" [Heading] OR "Eating disorder*" OR anorex* OR “anorexia nervosa” OR bulimia OR “binge-eating disorder” | N = 7 |
| AND | "Inpatients" [Heading] OR Inpatient OR “in hospital care” OR “in hospital treatment” OR treatment OR “mental health unit*” OR hospitalisation OR "acute admission" OR acute |  |
| AND | "Energy Intake" [Heading] OR "Food Intake" [Heading] OR “Caloric feeding protocol” OR “feeding protocol” OR “nutrition supplementation” OR “nutrition restoration” OR "enteral nutrition" OR “energy intake” OR "food intake" OR diet* |  |
| AND | "Refeeding Syndrome" [Heading] OR “refeeding syndrome” OR “hypophospha*” OR “phosphate” |  |
| AND | "Length of Stay" [Heading] OR "length of stay" OR "length in hospital" OR duration |  |
| Limiters: | 2012-2025; English; peer reviewed |  |
| **APA** | "Eating disorder*" OR anorex* OR “anorexia nervosa” OR bulimia OR “binge-eating disorder” | N = 7 |
| AND | Inpatient OR “in hospital care” OR “in hospital treatment” OR treatment OR “mental health unit*” OR hospitalisation OR "acute admission" OR acute |  |
| AND | “Refeeding syndrome” OR “hypophospha*” OR “phosphate” |  |
| AND | "Length of stay" OR "length in hospital" OR duration |  |
| Limiters: | 2012-2025; English; peer reviewed |  |
| **ProQuest** | "Eating disorders" [Search term] OR "Eating disorder*" OR anorex* OR “anorexia nervosa” OR bulimia OR “binge-eating disorder” | N = 477 |
| AND | "Inpatient care" [Search term] OR Inpatient OR “in hospital care” OR “in hospital treatment” OR treatment OR “mental health unit*” OR hospitalisation OR "acute admission" OR acute |  |
| AND | "Calories" [Search term] OR "Caloric feeding protocol" OR "feeding protocol" OR "nutrition supplementation" OR "nutrition restoration" OR "enteral nutrition" OR "energy intake" OR "food intake" OR diet* OR calori* |  |
| AND | "Length of stay" [Search term] OR "length of stay" OR "length in hospital" OR duration |  |
| AND | "refeeding syndrome" OR "hypophospha*" OR "phosphate" OR "electrolyte derangement" OR "refeeding" |  |
| Limiters: | 2012-2025, English, peer-reviewed, article [Scholarly journals], full text, subjects [‘anorexia’, ‘eating disorders’] |  |
| **Scopus** | "Eating disorder*" OR anorex* OR “anorexia nervosa” OR bulimia OR “binge-eating disorder” | N = 232 |
| AND | Inpatient OR “in hospital care” OR “in hospital treatment” OR treatment OR “mental health unit*” OR hospitalisation OR "acute admission" OR acute |  |
| AND | "Caloric feeding protocol" OR "feeding protocol" OR "nutrition supplementation" OR "nutrition restoration" OR "enteral nutrition" OR "energy intake" OR "food intake" OR diet* OR calori* |  |
| AND | "length of stay" OR "length in hospital" OR duration |  |
| AND | "refeeding syndrome" OR "hypophospha*" OR "phosphate" OR "electrolyte derangement" OR "refeeding" |  |
| Limiters: | 2012-2023, English, article [Scholarly journals] |  |
| Total records identified after database searching | | N = 734 |
| Total records after duplicates removed | | N = 503 |
