# Supplementary material for: Can higher calorie feeding protocols for eating disorder patients admitted to an adult hospital ward be safely implemented to reduce the length of stay? A scoping review
Source: J Eat Disord. 2026 Jul 30;14:260. doi: 10.1186/s40337-026-01669-9 (PMC13741680; doi:10.1186/s40337-026-01669-9)
Supplement: Supplementary file 2 [file 40337_2026_1669_MOESM2_ESM.docx]

### Supplementary 2: Inclusion and exclusion criteria (Population, Issue, Outcomes)

|  | **Inclusion** | **Exclusion** |
| --- | --- | --- |
| **Publication year** | 1^st^ Jan 2012 until 30^th^ June 2025 | Prior to 2012 |
| **Language** | English | Non-English |
| **Population** | Initiating feeding protocols for eating disorder patients admitted within an inpatient adult ward setting requiring nutritional restoration. | Paediatric wards |
| **Issue** | Commencing higher caloric refeeding (HCR) at a rate of ≥1500 calories/day for eating disorder patients admitted to an inpatient adult ward. | Commencing on lower calorie refeeding (LCR) at a rate of <1500 calories within 24 hours of inpatient admission. |
| **Outcomes** | Study mentioned at least one primary measure related to refeeding syndromes markers during hospital admission.   - Hypophosphatemia - Hypokalaemia - Hypomagnesemia - Peripheral oedema - Combination of the above   Secondary outcomes of interest   - Length of stay - Rate of weight gain and changes in admission - Discharge Body Mass Index (BMI) | Study did not report on any refeeding syndrome markers. |
